# An improved nonsingular adaptive super twisting sliding mode controller for quadcopter

**DOI:** 10.1371/journal.pone.0309098

**Published:** 2024-10-10

**Authors:** Nardos Belay Abera, Chala Merga Abdissa, Lebsework Negash Lemma

**Affiliations:** School of Electrical and Computer Engineering, Addis Ababa University, Addis Ababa, Ethiopia; University of Shanghai for Science and Technology, CHINA

## Abstract

This paper presents an improved nonsingular adaptive super twisting sliding mode control for tracking of a quadrotor system in the presence of external disturbances and uncertainty. The initial step involves developing a dynamic model for the quadrotor that is free from singularities, achieved through the utilization of the Newton-Quaternion formalism. Then, the super twisting algorithm is used to develop a novel sliding mode control that mitigates chattering. Particle Swarm Optimization (PSO) is employed for the adjustment of the controller gains. Moreover, to maintain stable control of the quadcopter, even in scenarios where the upper limit of disturbances is unknown, an adaptive rule grounded in Lyapunov stability is applied. Simulation results demonstrate that the proposed controller reduces tracking errors to 0.1% for roll, 0.05% for pitch, and 2.2% for altitude, outperforming other state-of-the-art sliding mode controllers. Additionally, the proposed controller effectively rejects disturbances, maintaining minimal steady-state errors of 0.01° for roll, 0.02° for pitch, and 0.001° for yaw, significantly better than conventional controllers. These results highlight tracking and disturbance rejection capabilities of the proposed controller, making its real-time implementation for quadrotor Unmanned Aerial Vehicles (UAVs) feasible.

## 1 Introduction

UAVs are autonomous aircraft that have been in existence for a while [1, 2]. Aerial photography, crop spraying in agriculture, and payload transportation are only a few examples of the applications of quadrotor UAVs [3, 4]. The control system of a quadrotor UAV serves as its central processing unit, responsible for ensuring the correct position and orientation during flight. Designing high performance controllers for quadcopters presents challenges due to factors such as time-varying environmental conditions and nonlinear coupled dynamics of the system [5].

The mathematical models of quadcopters constitute nonlinear systems. The system, controlled by four input signals, operates with six degrees of freedom (DOFs). This implies a strong interdependence among the dynamic states. Consequently, the control strategy for the quadcopter typically adopts a multi-loop approach, where an outer loop prioritizes position control, while an inner loop focuses on attitude control [6].

Several linear controllers, including H-infinity [7], and conventional Proportional Integral Derivative (PID) [8], have been proposed for controlling of quadcopter. However, these methods often become less effective when there is an external disturbances. Adaptive backstepping, as discussed by [9, 10], was developed to improve the tracking abilities of quadrotors. This study presents a control scheme that achieves stable control within a predetermined time frame, ensuring precise control of both the position and attitude of a quadrotor.

Hence, it is crucial to develop a robust controller to surmount these challenges. Sliding Mode Control (SMC) is well-known for its robustness in dynamic environments [11, 12]. The paper in [13], proposes a comprehensive examination of SMC techniques, emphasizing their robustness and effectiveness in managing the nonlinear dynamics and uncertainties inherent in robotic manipulators. The paper in [14], proposes distributed robust SMC for the autonomous formation flight of quadrotors. In [15], an experimental validation of a fixed-time nonlinear homogeneous sliding mode approach for multirotor tracking control is proposed. Consequently, classical SMC techniques are commonly employed across various UAV applications. However, the persistence of a discontinuous term in the control law leads to chattering, potentially resulting damage to electromechanical systems. In [16], various SMC techniques are examined with their applications in underactuated systems. The review highlights significant advancements and ongoing challenges in the field, especially focusing on efforts to reduce chattering a common issue in SMC and to enhance robustness and control accuracy. To address this issue, numerous enhancements have been suggested. In order to mitigate chattering, an approach known as higher-order sliding mode control (HOSMC) was introduced in [17]. In [18], the discontinuous control of SMC was replaced with continuous “saturation” or “sigmoid” characteristics. However, this adjustment no longer ensures that the trajectories of the sliding system reach the sliding surface due to the sigmoid function’s approximation, potentially reducing the accuracy of the control action. In [19], the review discusses the application of SMC in various motion control systems, providing explicit design parameters and highlighting the challenge of determining the controller gain.

In [20, 21], backstepping sliding mode algorithms have been developed for trajectory tracking of quadcopters. However, finding the controller’s parameters poses a challenge. In [22], a robust backstepping integral sliding mode control (RBISMC) technique designed for quadcopter flight control, and it compares this approach with fraction order integral sliding mode control (FOISMC). In [23], robust tracking control for quadrotor UAVs is proposed using neural network-based MRAC. In [24], a genetic algorithm-tuned super twisting sliding mode controller is proposed for maglev train suspension. In [25], position tracking control using an adaptive SMC technique provided. The methods mentioned above are frequently capable of addressing issues related to trajectory tracking control in environments with external uncertainties and disturbances. However, most of these approaches tend to be highly intricate, and face challenges in acquiring the controller gains.

The PSO algorithm represents a recent and successful approach for effectively and efficiently tuning gains across various systems and types of controllers [26, 27]. In [28], PSO algorithm, specifically PSO-PID, is utilized in quadcopters to minimize tuning efforts while achieving optimal outcomes.

In this paper, an improved Nonsingular adaptive super twisting sliding mode controller is developed to stabilize the disturbed quadrotor and guarantee precise tracking when disturbances occur. Specifically, PSO-based sliding manifolds are tailored for the attitude and position subsystems, aiming to achieve accurate trajectory tracking in the quadrotor system. The contributions of this research paper can be outlined as follows:

- A new design for an Improved Nonsingular Adaptive Super Twisting Sliding Mode Controller has been introduced, aiming to enhance robust tracking control for quadrotor UAVs by utilizing quaternion modeling,
- Utilizing the Newton-Quaternion formalism a quaternion-based dynamic model, free from singularities, is developed to enhance the effectiveness of the controller design.
- Through simulation, the proposed method has been developed and evaluated in the presence of external disturbances, demonstrating better performance compared to other state-of-the-art methods [29–31].
- PSO approach has been executed to improve controller performance by tuning the sliding manifold gains which in turn increases tracking performance,
- An adaptation gain has been adopted to tune the gains of super twisting SMC.

The paper proceeds as follows: Section 2, elaborates on the mathematical model of quadcopter utilizing quaternions. Section 3, discusses the proposed controller and analyse stability. Section 4, presents the simulation results. Lastly, concluding remarks are given in Section 5.

## 2 Mathematical model of quadcopter

### 2.1 Quaternion based modeling

Various assumptions are made in the literature to simplify the modeling of the quadcoper. Universal modeling assumptions taken from various literature [32, 33] and adopted in these papers are presented here as follows:

- The propellers of quadcopter is considered rigid.
- The center of gravity (COG) of the vehicle coincides with the origin of the body’s coordinate system.
- The quadcopter has a symmetric structure and the inertia matrix around this symmetry is diagonal.
- Thrust is proportional to the square of rotor speed.
- Torque is proportional to the square of rotor speed.

### 2.2 Reference frames alignment

A reference body is a conceptual body of coordinates that can be used to describe the positioning and motion of objects. The quadcopter function in two coordinate frames: the inertial frame and the body frame [34].

The orientation of the coordinate system is established by applying the right-hand rule. The positive direction of the *x*–axis is indicated by the thumb, the positive direction of the *y*–axis by the first finger, and the positive direction of the *z*–axis by the middle finger. Two coordinate frames are utilized due to the quadcopter array of sensors. Sensors like gyroscopes and accelerometers provide readings with respect to the body frame, whereas sensors such as Global Positioning System (GPS) and magnetometers provide readings relative to the inertial frame. The body frame is linked to the quadcopter, with its origin positioned at COG.

**Fig 1:**
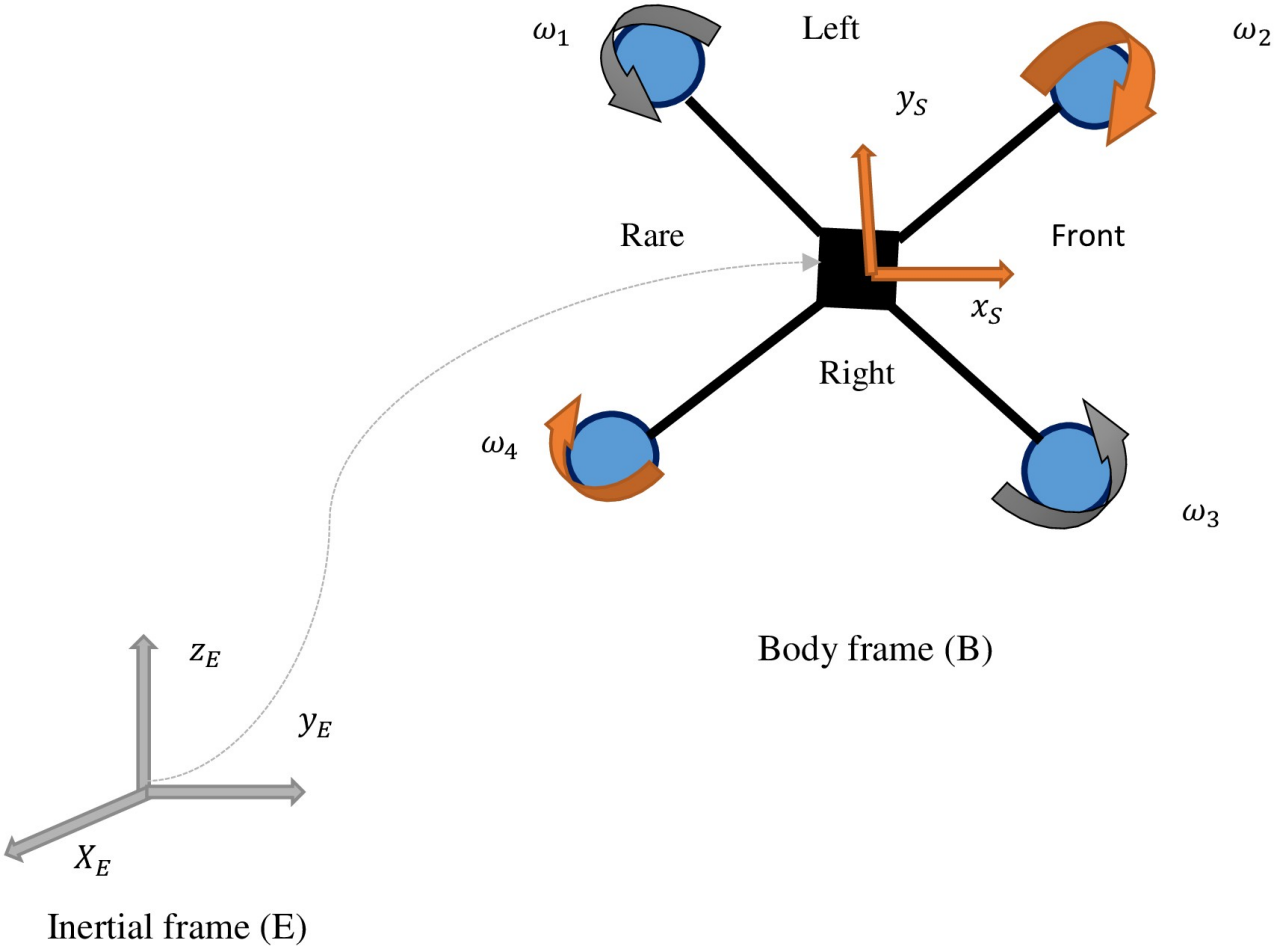
Alignment of quadcopter reference frames, showing the alignment between body and inertial frames.

Fig 1, shows the reference frame alignment of the quadcopter. A quadcopter orientation or attitude in three-dimensional space is represented by a rotation matrix. The rotation matrix in the context of a quadcopter is used to define the rotation of the quadcopter body frame with respect to the inertial frame [35]. The quaternion method is employed for transformation to circumvent the challenges commonly associated with Euler angles, such as geometric singularities, nonlinearity, and computational complexity [36, 37].

A four-dimensional mathematical construct is called a quaternion, encompassing both scalar and vector components. a scalar components *q*_*o*_, and vector components *q* = *q*_0_ + *q*_1_*i* + *q*_2_*j* + *q*_3_*k*, where *q*_0_, *q*_1_, *q*_2_ and *q*_3_ are real numbers, and *i*, *j*, and *k* are the quaternion units [38].

A unit quaternion is a straightforward mathematical representation utilized to specify orientations in three-dimensional space. Hence, the dynamic model of the quadrotor is formulated based on the principle of unit quaternions. The process of calculating a coordinate transformation using quaternion rotation between distinct reference frames is articulated as [39]:

$$\begin{array}{c} Y=q⊗Y^{T}⊗q^{-1} \end{array}$$ (1)

where *Y*^*T*^ represents the vector undergoing rotation, while *Y* denotes the vector after rotation has been applied. As per Eq 1, when you multiply two or more rotation quaternions, you obtain a new rotation quaternion. This new quaternion represents the combined effect of each individual rotation performed by each quaternion independently. In order to move vectors from one axis system to another, it’s necessary to convert them into pure quaternions where the scalar component is zero. Eq 1, can be reformulated to yield a rotation matrix using quaternions, facilitating the transition of variables from the Inertial Frame(IF) to the Body Frame (BF).

$$\begin{array}{c} {}[\begin{array}{c} 0\\ R_{b} \end{array}]=q⊗[\begin{array}{c} 0\\ R_{i} \end{array}]⊗q^{*} \end{array}$$ (2)

where *R*_*b*_ = [*x*_*b*_, *y*_*b*_, *z*_*b*_] represents the position vector in BF coordinate system and *R*_*i*_ = [*x*_*i*_, *y*_*i*_, *z*_*i*_] represents the position vector in the IF coordinate system. Eq 2, can be rearranged as follows to calculate $R_{E}^{B}(\text{q})$:

$$R_{E}^{B}=[\begin{array}{ccc} q_{0}^{2}+q_{1}^{2}+q_{2}^{2}-q_{3}^{2} & 2\left(q_{1}q_{2}-q_{0}q_{3}\right) & 2\left(q_{1}q_{3}+q_{0}q_{2}\right)\\ 2\left(q_{1}q_{2}+q_{0}q_{3}\right) & q_{0}^{2}-q_{1}^{2}+q_{2}^{2}-q_{3}^{2} & 2\left(q_{2}q_{3}-q_{0}q_{1}\right)\\ 2\left(q_{1}q_{3}-q_{0}q_{2}\right) & 2\left(q_{2}q_{3}-q_{0}q_{1}\right) & q_{0}^{2}-q_{1}^{2}-q_{2}^{2}+q_{3}^{2} \end{array}]$$ (3)

Projecting BF rates onto IF using a rate transformation matrix:

$$\begin{array}{c} \dot{q}=\frac{1}{2}[\begin{array}{cccc} q_{0} & -q_{1} & -q_{2} & -q_{3}\\ q_{1} & q_{0} & q_{3} & q_{2}\\ q_{2} & q_{3} & q_{0} & -q_{1}\\ q_{3} & -q_{2} & q_{1} & q_{o} \end{array}][\begin{array}{c} 0\\ p\\ q\\ r \end{array}] \end{array}$$ (4)

### 2.3 Translational motion of a quadrotor

The quadrotor translational dynamics in the IF can be described as follows:

$$\begin{array}{c} \sum F^{E}=F_{T}^{E}+F_{g}^{E}=m\vec{a} \end{array}$$ (5)

where $F_{T}^{E}$ trust force in IF, $F_{g}^{E}$ gravitational force in IF, *m* mass $\vec{a}$ is linear acceleration. Using Newton second law: $F=m\vec{a}$ the transitional forces with respect to earth frame can be described:

$$\begin{array}{c} \left[\begin{array}{c} m\ddot{x}\\ m\ddot{y}\\ m\ddot{z} \end{array}]=[\begin{array}{c} 2\left(q_{1}q_{3}-q_{0}q_{2}\right)F_{T}^{B}\\ 2\left(q_{2}q_{3}+q_{0}q_{1}\right)F_{T}^{B}\\ 2\left(2\left(q_{0}^{2}+q_{3}^{2}\right)-1\right)F_{T}^{B} \end{array}]+[\begin{array}{c} 0\\ 0\\ -mg \end{array}\right] \end{array}$$ (6)

where *F*_*g*_ = *mg* is a force of gravitation acting, *m* is mass, $\ddot{x},\ddot{y},\ddot{z}$ are the linear accelerations, and *g* is the gravity of the quadrotor in the inertial frame (IF). The equation governing the dynamics of translational motion relative to the earth frame is stated as follows:

$$\begin{array}{c} \ddot{x}=2\left(q_{1}q_{3}-q_{0}q_{2}\right)\frac{U_{1}}{m} \end{array}$$ (7)

$$\begin{array}{c} \ddot{y}=2\left(q_{2}q_{3}+q_{0}q_{1}\right)\frac{U_{1}}{m} \end{array}$$ (8)

$$\begin{array}{c} \ddot{z}=2\left(2\left(q_{0}^{2}+q_{3}^{2}\right)-1\right)\frac{U_{1}}{m}-g \end{array}$$ (9)

where *U*_1_ is the overall force of thrust employed in altitude control.

### 2.4 Rotational motion of quadrotor

**The gyroscopic effect**. The propellers’ rotation causes the gyroscopic effect in a quadcopter. The gyroscopic effect is caused by the torque, or rotational force, that the propellers produce as they spin [41].

$$\begin{array}{c} M_{gyro}=-\sum_{k=1}^{4}J_{tp}(\begin{array}{c} p\\ q\\ r \end{array})\times (\begin{array}{c} 0\\ 0\\ 1 \end{array}){\left(-1\right)}^{K}w_{k} \end{array}$$ (10)

where *J*_*tp*_ represents the combined rotational moment of inertia about the propeller axis.

$$\begin{array}{c} \sum \tau_{i}=J\alpha =[U_{2},U_{3},U_{4}{]}^{\prime }-M_{\text{gyro}\;} \end{array}$$ (11)

$$\begin{array}{c} \dot{\omega }=J^{-1}[[U_{2},U_{3},U_{4}{]}^{\prime }-\omega \times \left(J\omega \right)-M_{\text{gyro}\;}] \end{array}$$ (12)

where $\alpha =\dot{\omega }={\left[\dot{p},\dot{q},\dot{r}\right]}^{\prime }$ are angular accelerations; *J* represents inertia matrix; *M*_gyro_ is a gyroscopic moment: *U*_2_, *U*_3_, *U*_4_ are moments and *ω* represents angular speed in BF. By reorganizing Eq 12, the rotational dynamics of the quadrotor derived as.

$$\begin{array}{c} \dot{p}=\frac{U_{2}}{I_{x}x}-\frac{qr(I_{zz}-I_{y}y)}{I_{x}x}+\frac{M_{\text{gyro}\;}q}{I_{x}x} \end{array}$$ (13)

$$\begin{array}{c} \dot{q}=\frac{U_{3}}{I_{yy}}-\frac{rp(I_{xx}-I_{zz})}{I_{yy}}+\frac{M_{\text{gyro}\;}p}{I_{xx}} \end{array}$$ (14)

$$\begin{array}{c} \dot{q}=\frac{U_{4}}{I_{zz}}-\frac{pq(I_{yy}-I_{xx})}{I_{zz}} \end{array}$$ (15)

The dynamics description in the IF is achieved by employing the transfer matrix as shown below

$$\begin{array}{c} \dot{q_{0}}=\frac{1}{2}\left(pq_{1}+qq_{2}+rq_{3}\right) \end{array}$$ (16)

$$\begin{array}{c} \dot{q_{1}}=\frac{1}{2}\left(pq_{0}+rq_{2}-qq_{3}\right) \end{array}$$ (17)

$$\begin{array}{c} \dot{q_{2}}=\frac{1}{2}\left(qq_{0}+pq_{3}-rq_{1}\right) \end{array}$$ (18)

$$\begin{array}{c} \dot{q_{3}}=\frac{1}{2}\left(rq_{0}+qq_{1}-pq_{2}\right) \end{array}$$ (19)

The complete dynamics of quadrotor flight are outlined as follows:

$$\begin{array}{c} \left\{ \begin{array}{cc} \ddot{x} & =2\left(q_{1}q_{3}-q_{0}q_{2}\right)U_{1}/m\\ \ddot{y} & =2\left(q_{2}q_{3}+q_{0}q_{1}\right)U_{1}/m\\ \ddot{z} & =2\left(2\left(q_{0}^{2}+q_{3}^{2}\right)-1\right)U_{1}/m\\ \dot{p} & =\frac{U2}{I_{xx}}-\frac{qr(I_{zz}-I_{yy})}{I_{xx}}+\frac{M_{\text{gyro}\;}q}{I_{xx}}\\ \dot{q} & =\frac{U3}{I_{yy}}-\frac{rp(I_{xx}-I_{zz})}{I_{yy}}+\frac{M_{\text{gyro}\;}p}{I_{xx}}\\ \dot{q} & =\frac{U4}{I_{zz}}-\frac{pq(I_{yy}-I_{xx})}{I_{zz}}\\ \dot{q_{0}} & =\frac{1}{2}\left(pq_{1}+qq_{2}+rq_{3}\right)\\ \dot{q_{1}} & =\frac{1}{2}\left(pq_{0}+rq_{2}-qq_{3}\right)\\ \dot{q_{2}} & =\frac{1}{2}\left(qq_{0}+pq_{3}-rq_{1}\right)\\ \dot{q_{3}} & =\frac{1}{2}\left(rq_{0}+qq_{1}-pq_{2}\right) \end{array}\right. \end{array}$$ (20)

The following control allocation equations were developed based on the relationship between propeller rotational velocity and induced torque:

$$\begin{array}{c} \left\{ \begin{array}{c} \omega_{1}=\sqrt{\left(\frac{U_{1}}{4k_{f}}+\frac{U_{2}}{4k_{f}\ell }+\frac{U_{3}}{4k_{f}\ell }-\frac{U_{4}}{4d}\right)}\\ \omega_{2}=\sqrt{\left(\frac{U_{1}}{4k_{f}}-\frac{U_{2}}{4k_{f}\ell }+\frac{U_{3}}{4k_{f}\ell }+\frac{U_{4}}{4d}\right)}\\ \omega_{3}=\sqrt{\left(\frac{U_{1}}{4k_{f}}-\frac{U_{2}}{4k_{f}\ell }-\frac{U_{3}}{4k_{f}\ell }-\frac{U_{4}}{4d}\right)}\\ \omega_{4}=\sqrt{\left(\frac{U_{1}}{4k_{f}}+\frac{U_{2}}{4k_{f}\ell }-\frac{U_{3}}{4k_{f}\ell }+\frac{U_{4}}{4d}\right)} \end{array}\right. \end{array}$$

The total net propeller angular speed, denoted as *ω*_*T*_, is determined as follows

$$\omega_{T}=-\omega_{1}+\omega_{2}-\omega_{3}+\omega_{4}$$

To convert from quaternion to euler angles by employing a conventional set of Euler angles in the following manner [42]:

$$\left[\begin{array}{c} \phi \\ \theta \\ \psi \end{array}]=[\begin{array}{c} \text{arctan}\;2(2(q_{o}q_{1}+q_{2}q_{3}),{q_{o}}^{2}-{q_{1}}^{2}-{q_{2}}^{2}+{q_{3}}^{2})\\ \text{arcsin}\;(2(q_{o}q_{2}-q_{3}q_{1}))\\ \text{arctan}\;2(2(q_{o}q_{3}+q_{1}q_{2}),{q_{o}}^{2}+{q_{1}}^{2}-{q_{2}}^{2}-{q_{3}}^{2}) \end{array}\right]$$

## 3 Design of controller for quadcopter

Despite having four rotors, the translational dynamics of quadcopters tend to be underactuated. This indicates that they are not equipped with enough control inputs to directly regulate every DOF, particularly lateral motion. The system is enhanced by incorporating virtual control inputs, aiming to ensure that each output of the quadrotor aligns with a predetermined trajectory [43]. The following can be used to obtain the virtual control inputs:

$$\begin{array}{c} \left\{ \begin{array}{c} U_{x}=2(q_{1_{d}}q_{3_{d}}-q_{o_{d}}q_{2_{d}})U_{1}\\ U_{y}=2(q_{2_{d}}q_{3_{d}}+q_{o_{d}}q_{1_{d}})U_{1}\\ U_{z}=[2({q_{o_{d}}}^{2}+{q_{3_{d}}}^{2})-1]U_{1}-mg \end{array}\right. \end{array}$$

where the parameters *U*_*x*_, *U*_*y*_, and *U*_*z*_ denote the virtual control inputs for guiding the quadrotor position along the *x*, *y*, and *z* axes respectively. The quadrotor translational dynamics can be represented as:

$$\begin{array}{c} \ddot{x}=\frac{U_{x}}{m} \end{array}$$ (21)

$$\begin{array}{c} \ddot{y}=\frac{U_{y}}{m} \end{array}$$ (22)

$$\begin{array}{c} \ddot{z}=\frac{U_{z}}{m} \end{array}$$ (23)

To design controller for a second order system, the rotational dynamics of a quadrotor are rewritten in quaternion using the small quaternion approach.

$$\left[\begin{array}{c} {\dot{q}}_{o}\\ {\dot{q}}_{1}\\ {\dot{q}}_{2}\\ {\dot{q}}_{3} \end{array}]=\frac{1}{2}[\begin{array}{cccc} 1 & 0 & 0 & 0\\ 0 & 1 & 0 & 0\\ 0 & 0 & 1 & 0\\ 0 & 0 & 0 & 1 \end{array}][\begin{array}{c} 0\\ p\\ q\\ r \end{array}]=\frac{1}{2}[\begin{array}{c} 0\\ p\\ q\\ r \end{array}\right]$$

Hence,

$$\left[\begin{array}{c} p\\ q\\ r \end{array}]=2[\begin{array}{c} {\dot{q}}_{1}\\ {\dot{q}}_{2}\\ {\dot{q}}_{3} \end{array}\right]$$

$$\left[\begin{array}{c} \dot{p}\\ \dot{q}\\ \dot{r} \end{array}]=2[\begin{array}{c} {\ddot{q}}_{1}\\ {\ddot{q}}_{2}\\ {\ddot{q}}_{3} \end{array}\right]$$

Finally, the second-order rotational dynamics of the quadrotor can be expressed using quaternions as follows:

$$\left\{ \begin{array}{c} {\ddot{q}}_{1}=\frac{1}{J_{xx}}(\frac{1}{2}U_{2})\\ {\ddot{q}}_{2}=\frac{1}{J_{yy}}(\frac{1}{2}U_{3})\\ {\ddot{q}}_{3}=\frac{1}{J_{zz}}(\frac{1}{2}U_{4}) \end{array}\right.$$

The state vector is composed of two components: orientation control, expressed as $\left[q_{1},{\dot{q}}_{1},q_{2},{\dot{q}}_{2},q_{3},{\dot{q}}_{3}\right]$ and position control, expressed as $\left[x,\dot{x},y,\dot{y},z,\dot{z}\right]$.

### 3.1 Design of a quadrotor position controller

The first step to design an improved nonsingular adaptive super twisting sliding mode controller is designing sliding manifold of SMC. SMC enables the design of controllers for both linear and nonlinear processes. The concept of the SMC technique is to force the command signal to move to the sliding surface and stay on that surface after the command signal has been reached. In order to guarantee that the system states stay on the sliding surface, the control law is then designed. The two phases of SMC are [44]:

**Reaching phase**: This phase involves designing the control law to drive the system’s state trajectories towards a predefined sliding surface. The sliding surface is a manifold where the control objectives are met. During the reaching phase, a discontinuous control law is applied to force the system to reach the sliding surface despite the presence of uncertainties and disturbances.

**Sliding phase**: Once the system state reaches the sliding surface, the control switches to ensure that the state remains on this surface for all times. This phase is characterized by the system’s dynamics being confined to the sliding surface, designed to be invariant. The control law in this phase maintain the system on the sliding surface, ensuring robust performance against perturbations and uncertainties.

**Fig 2:**
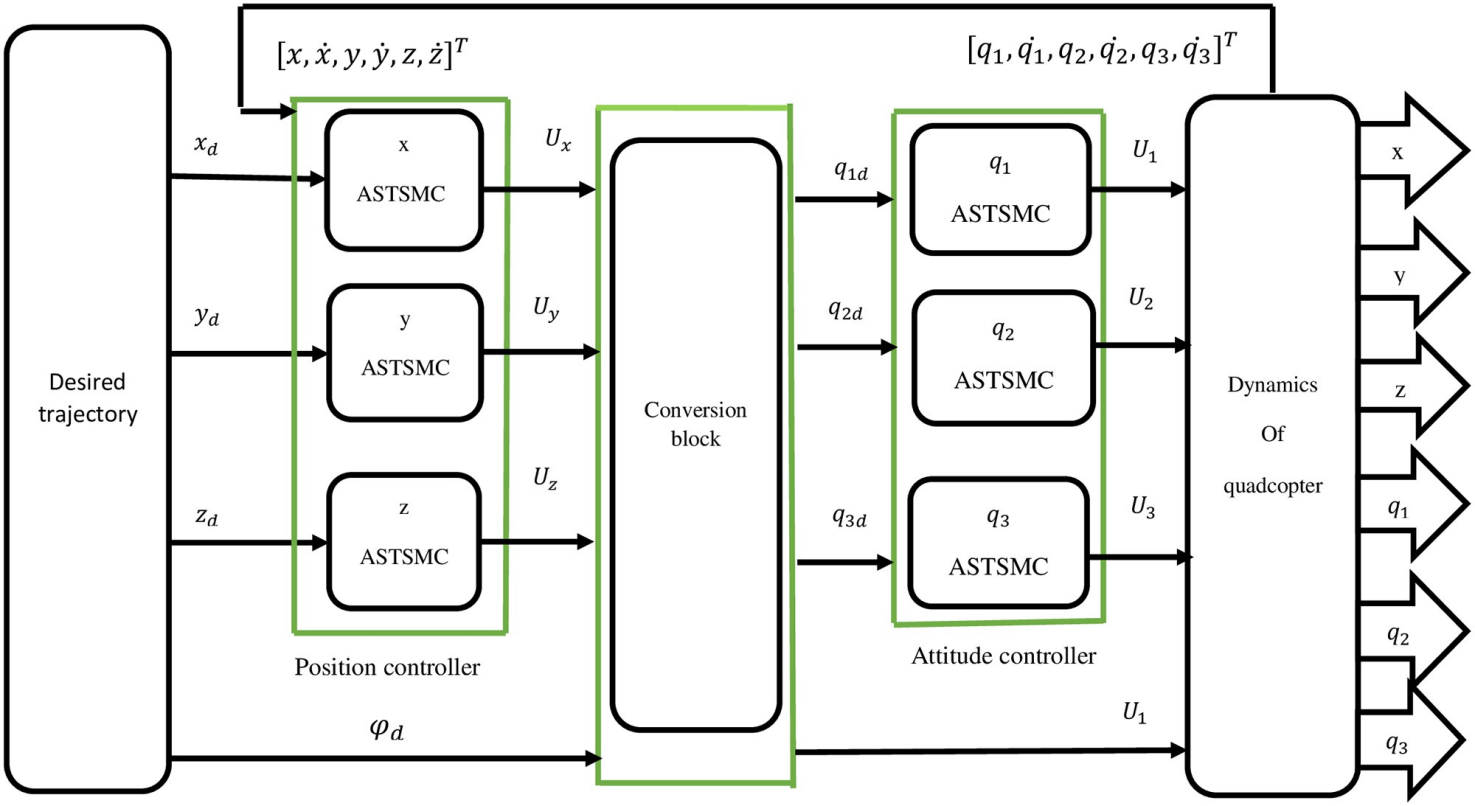
Controller architecture for quadrotor, including position, altitude, and virtual control input designs.

These phases are crucial for ensuring that SMC achieves its robustness and performance despite the presence of uncertainties and external disturbances. Fig 2, illustrates the complete controller architecture, including the position and altitude controllers, as well as the virtual control input.

**Design sliding manifold**. The following procedures should be taken to design a sliding manifold.

1. Determine the quadcopter desired trajectory.
  $$\begin{array}{c} \left[\begin{array}{c} e_{x}\\ e_{y}\\ e_{z} \end{array}]=[\begin{array}{c} x_{d}-x\\ y_{d}-y\\ z_{d}-z \end{array}],[\begin{array}{c} {\dot{e}}_{x}\\ {\dot{e}}_{y}\\ {\dot{e}}_{z} \end{array}]=[\begin{array}{c} {\dot{x}}_{d}-\dot{x}\\ {\dot{y}}_{d}-\dot{y}\\ {\dot{z}}_{d}-\dot{z} \end{array}\right] \end{array}$$ (24)
2. Design a sliding manifold, a function that measures the difference between the quadcopter actual and desired states. Designing the sliding surface in accordance with the dynamics of the error is the first stage in minimizing the difference between the desired and the actual value, and Stoline and Li proposed the general form of the following equation in [45]:
  $$\begin{array}{c} \sigma ={\left(\lambda +\frac{d}{dt}\right)}^{p}e \end{array}$$ (25)
  where *e* is the tracking error, defined as for *z*-axis *e*(*x*) = *z*_*d*_ − *z* (the error between the desired trajectory and the actual trajectory in the x-axis); λ and *c* is a positive constant that interprets the dynamics of the surface and *p* = *n* − 1, *n* is relative degree between output and input. Based on Eq 25, position controller sliding manifold can be designed:
  $$\begin{array}{c} \left\{ \begin{array}{c} \sigma_{x}=\lambda_{x}e_{x}+c_{x}{\dot{e}}_{x}\\ \sigma_{y}=\lambda_{x}e_{y}+c_{y}{\dot{e}}_{y}\\ \sigma_{z}=\lambda_{x}e_{z}+c_{z}{\dot{e}}_{z} \end{array}\right. \end{array}$$ (26)
3. Design control law and tune the gain of sliding surface using PSO.

### 3.2 Design super twisting sliding mode controller

STSMC is given by the equation below:

$$\begin{array}{c} U_{st}=-\alpha \sqrt{|\sigma |}sign\left(\sigma \right)-v \end{array}$$ (27)

$$\begin{array}{c} \dot{v}=\frac{\beta }{2}sign\left(\sigma \right) \end{array}$$ (28)

The total control law (*U*) calculated by summing the equivalent and supertwisting control law:

$$\begin{array}{c} U=U_{equ}+U_{st} \end{array}$$ (29)

where *U*_*equ*_ is equivalent controller and *U*_*st*_ is super twisting controller.

**Fig 3:**
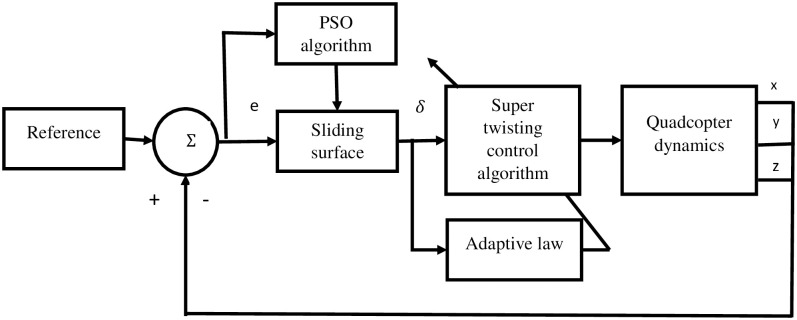
Diagram of position controller for maintaining quadrotor trajectory along x, y, and z axes.

Fig 3, illustrates the position controller diagram designed for the quadcopter. The controller focuses on maintaining the desired trajectory by controlling the quadcopter position in the x, y, and z axes.

After selecting sliding surface the next step is design equivalent control law and this law ensure the system remains in sliding mode during operation. Equivalent control law can be designed by making ${\dot{\sigma }}_{x},{\dot{\sigma }}_{y},{\dot{\sigma }}_{z}=0$.

The virtual controller (*U*_*x*_, *U*_*y*_, *U*_*z*_) which means that the three common input will control motion along x, y and z and these virtual control are given by equation.

$$\begin{array}{c} \left\{ \begin{array}{cc} U_{x} & =\frac{\lambda_{x}}{C_{x}}\dot{e_{x}}+\ddot{x_{d}}-\alpha_{x}\sqrt{|\sigma_{x}|}sign\left(\sigma_{x}\right)-v_{x}\\ U_{y} & =\frac{\lambda_{y}}{C_{y}}\dot{e_{y}}+\ddot{y_{d}}-\alpha_{y}\sqrt{|\sigma_{y}|}sign\left(\sigma_{y}\right)-v_{y}\\ U_{z} & =\frac{\lambda_{z}}{C_{z}}\dot{e_{z}}+\ddot{z_{d}}-\alpha_{z}\sqrt{|\sigma_{z}|}sign\left(\sigma_{z}\right)-v_{z} \end{array}\right. \end{array}$$ (30)

where *α*, *β* are the gain of super twisting algorithm and $\dot{v}=-\frac{\beta }{2}sign\left(\sigma \right)$.

**Theorem 1**. If the quadrotor position system 20, is taken into consideration, together with the PSO based sliding manifold 26, control laws 30, and tracking errors 24, asymptotically, the closed loop tracking error can approach zero.

The control law must ensure that $\dot{V(z)}<0$ and the system state variables *V*(*z*) > 0.

Proof. Lyapunov function is a positive scalar function. The Lyapunov function is defined as $V_{z}=\frac{1}{2}\sigma^{T}\sigma$. The control law must ensure that $\dot{V(z)}<0$ and the system state variables *V*(*z*) > 0.

$$\begin{array}{cc} {\dot{V}}_{z} & =\sigma_{z}\dot{\sigma_{z}}<0\\ \dot{V_{z}} & =\sigma_{z}\left(\lambda_{z}\dot{e_{z}}+c_{z}\left(\ddot{z_{d}}-\ddot{z}\right)\right) \end{array}$$

To substitute the value of $\ddot{z}$, use the virtual control law.

$$\begin{array}{cc} & =\lambda_{z}\dot{e_{z}}+c_{z}\left(\ddot{z_{d}}-\alpha_{z}\sqrt{|\sigma_{z}|}sign\left(\sigma_{z}\right)-\int \beta_{z}sign\left(\sigma_{z}\right)dt\right)\\ \dot{V_{z}} & =\sigma_{z}\left(\lambda_{z}\dot{e_{z}}+c_{z}\ddot{z_{d}}-\lambda_{z}\dot{e_{z}}-c_{z}\ddot{z_{d}}-\alpha_{z}\sqrt{|\sigma_{z}|}sign\left(\sigma_{z}\right)-\int \beta_{z}sign\left(\sigma_{z}\right)dt\right)\\ \dot{V_{z}} & =\sigma_{z}\left(-\alpha_{z}\sqrt{|\sigma_{z}|}sign\left(\sigma_{z}\right)-\int \beta_{z}sign\left(\sigma_{z}\right)dt\right)<=0 \end{array}$$ (31)

To make the Lyapunov function valid, the gains *α*_*z*_, *β*_*z*_ should be above positive. The position subsystem uses the proposed controller, and the stability of the loop is demonstrated using the Lyapunov principle.

The virtual control in Eq 30, used to determine desired quaternion value. when *x*, *y* and *z* approach *x*_*d*_, *y*_*d*_, and *z*_*d*_ as time reaches the sliding surface *σ*_*x*_ = 0, *σ*_*y*_ = 0, *σ*_*z*_ = 0 and the designed controller derives the actual value to the desired position. The desired quaternion value can also be calculated by rearranging the virtual control inputs.

$$\begin{array}{c} U_{1}=\sqrt{U_{x}^{2}+U_{y}^{2}+{\left(U_{z}+mg\right)}^{2}} \end{array}$$ (32)

$$\begin{array}{c} q_{0d}=\frac{1}{2}\sqrt{\frac{U_{z}+mg}{U_{1}}-2sin^{2}\left(\psi_{d}/2\right)+1} \end{array}$$ (33)

$$\begin{array}{c} q_{1d}=sin\left(\psi_{d}/2\right)U_{x}+\frac{1}{\sqrt{2}}U_{y}\sqrt{\frac{U_{z}+mg}{U_{1}}-2sin^{2}\left(\psi_{d}/2\right)+1} \end{array}$$ (34)

$$\begin{array}{c} q_{2d}=\frac{sin\left(\psi_{d}/2\right)U_{y}-\frac{1}{\sqrt{2}}U_{x}\sqrt{\frac{U_{z}+mg}{U_{1}}-2sin^{2}\left(\psi_{d}/2\right)+1}}{U_{z}+mg+U_{1}} \end{array}$$ (35)

$$\begin{array}{c} q_{3d}=sin\left(\frac{\psi_{d}}{2}\right) \end{array}$$ (36)

### 3.3 Altitude controller design

**Fig 4:**
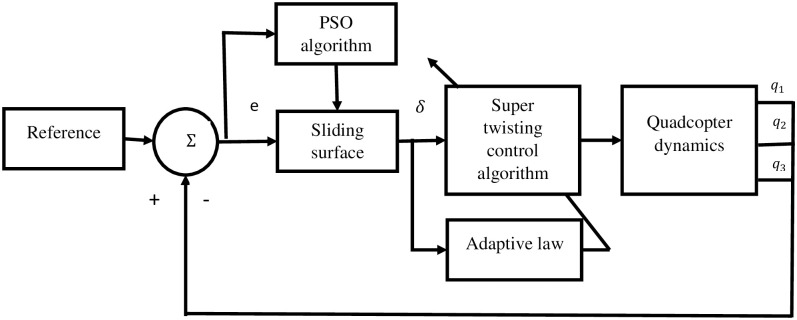
Altitude controller diagram for maintaining desired quadrotor altitude.

Fig 4, depicts the altitude controller diagram responsible for maintaining the quadcopter desired altitude. The controller operates by generating control input signals based on the reference inputs provided by the conversion blocks.

By generating the input control signal (*U*_2_, *U*_3_, *U*_4_), the adaptive super twisting sliding mode control maintains the tracking errors of the attitude subsystem. The tracking errors are defined:

$$\begin{array}{c} \left[\begin{array}{c} e_{q1}\\ e_{q2}\\ e_{q3} \end{array}]=[\begin{array}{c} q_{1d}-q_{1}\\ q_{2d}-q_{2}\\ q_{3d}-q_{3} \end{array}\right] \end{array}$$ (37)

Like position control design to design altitude controller first step is designing of sliding surface:

$$\begin{array}{c} \left\{ \begin{array}{cc} \sigma_{q1} & =\lambda_{q1}e_{q1}+c_{q1}{\dot{e}}_{q1}\\ \sigma_{q2} & =\lambda_{q2}e_{q2}+c_{q2}{\dot{e}}_{q2}\\ \sigma_{q3} & =\lambda_{q3}q_{3}+c_{q3}{\dot{e}}_{q3} \end{array}\right. \end{array}$$ (38)

**Theorem 2**. If the quadrotor attitude system 20, is designed with PSO-based sliding surfaces 38, the closed loop system’s tracking errors 37, can asymptotically converge to zero.

Proof. The Lyapunov function is stated as $V_{q1}=\frac{1}{2}\sigma^{T}\sigma$. with *V*(_*q*1_) > 0 and the control law requiring $\dot{V}{(}_{q1})<0$.

$$\begin{array}{cc} V_{q1} & =\frac{1}{2}\sigma_{q1}^{T}\sigma_{q1}\\ {\dot{V}}_{q1} & =\sigma_{q1}\dot{\sigma_{q1}}<=0\\ {\dot{V}}_{q1} & =\sigma_{q1}\left(\lambda_{q1}\dot{e_{q1}}+c_{q1}\left({\ddot{q}}_{1d}-\ddot{q_{1}}\right)\right)\\ {\dot{V}}_{q1} & =\sigma_{q1}\left(\lambda_{q1}\dot{e_{q1}}+c_{q1}\ddot{q_{1d}}-c_{q1}\left(\frac{I_{yy}-I_{zz}}{I_{xx}}\dot{q_{1}}\dot{q_{3}}-\frac{J_{t}}{I_{xx}}\dot{q_{1}}\omega \right)\right)\\ U_{2} & =I_{xx}\left(\frac{\lambda_{q1}}{c_{q1}}\dot{e_{q1}}+\ddot{q_{1d}}-\left(\frac{I_{yy}-I_{zz}}{I_{xx}}\dot{q_{1}}\dot{q_{3}}-\frac{J_{t}}{I_{xx}}\dot{q_{1}}\omega +U_{st}\right)\right)\\ {\dot{V}}_{q1} & =\sigma_{q1}\left(-\alpha_{q1}\sqrt{|\sigma_{q1}|}sign\left(\sigma_{q1}\right)-\int \beta_{q1}sign\left(\sigma_{q1}\right)dt\right)<=0 \end{array}$$

Consequently, the altitude tracking errors will asymptotically approach zero.

$$\left\{ \begin{array}{c} U_{2}=I_{xx}[\frac{\lambda_{q1}}{c_{q1}}{\dot{e}}_{q1}+{\ddot{q}}_{1d}-(\frac{I_{y}-I_{z}}{I_{xx}}{\dot{q}}_{1}{\dot{q}}_{3}-\frac{J_{t}}{I_{xx}}{\dot{q}}_{1}\omega_{T})]-\alpha_{q1}\sqrt{|\sigma_{q1}|}\text{sign}\left(\sigma_{q1}\right)+v_{q1}\\ U_{3}=I_{yy}[\frac{\lambda_{q2}}{c_{q2}}e_{q2}+{\ddot{q}}_{2d}-(\frac{I_{xz}-I_{xz}}{I_{yy}}{\dot{q}}_{2}{\dot{q}}_{3}+\frac{J_{t}}{I_{yy}}{\dot{q}}_{2}\omega_{T})]-\alpha_{q2}\sqrt{|\sigma_{q2}|}\text{sign}\left(\sigma_{q2}\right)+v_{q2}\\ U_{4}=I_{zz}[\frac{\lambda_{q3}}{c_{q3}}e_{q3}+{\ddot{q}}_{3d}-(\frac{I_{xx}-I_{yy}}{I_{xz}}{\dot{q}}_{1}{\dot{q}}_{2})]-\alpha_{q3}\sqrt{|\sigma_{q3}|}\text{sign}\left(\sigma_{q3}\right)+v_{q3}\\ \dot{v}=-\frac{\beta }{2}\text{sign}\left(\sigma \right) \end{array}\right.$$

### 3.4 SMC gain tuning

The particle swarm approach was developed by the movements of birds and swimming fish, the notion for the optimization of nonlinear systems was suggested by [46], and further developed in [47]. PSO is very simple and efficient method to adjust gain for controllers when compared to other optimization techniques [27]. In this research, a PSO is chosen to automatically adjust the quadcopter altitude and attitude control gain of SMC controller. The various parameters that make up this fitness function are taken from trajectory monitoring and regulation of the coupled and nonlinear dynamics of a quadcopter. Every particle’s position is associated with SMC. Each particle’s speed in the two simulations is corresponding to this parametrized change. Every particle and variable has its position and velocity initialized at random. Every particle travels across the search space, and every particle’s effectiveness is assessed on a regular basis.

**Fig 5:**
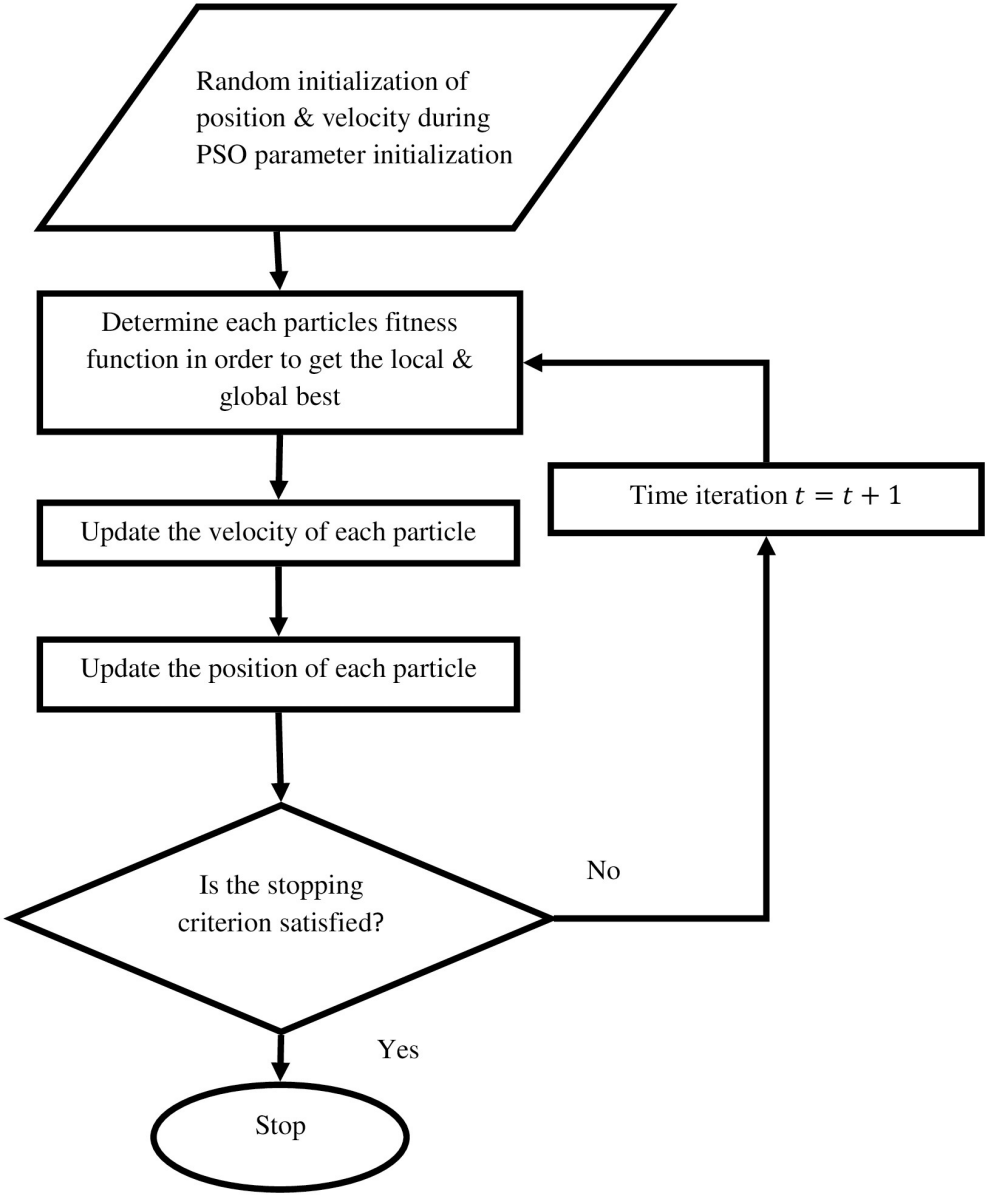
Flowchart of PSO algorithm used for tuning quadrotor sliding surface parameters.

The PSO algorithm is computationally efficient, conceptually straightforward, and simple to apply. Fig 5, presents the flowchart of PSO algorithm, which is employed to fine-tune the control parameters of the quadcopter sliding surface. The PSO algorithm iteratively optimizes the sliding surface gains. The flowchart outlines the key steps of the PSO process, from initializing the particles’ positions and velocities to evaluating their performance and updating their positions based on the best-performing particles.

### 3.5 Fitness function

Using the proposed approach, a performance index is used to quantify the optimization performance of the quadrotor’s responses. The purpose of the performance index, sometimes referred to as the fitness function, is to reduce the differences between the quadrotor’s controllable and desired output responses. The goal of these papaer is to reduce quadcopter trajectory tracking error in terms of both position and orientation. Systems constructed using Integral Time Absolute Error (ITAE) have minor overshoots and minimal damped oscillations. This paper utilized the ITAE performance index.

$$ITAE=\int_{0}^{\infty }t\left|e\left(t\right)\right|dt$$

### 3.6 Adaptive super twisting sliding mode control design

**Control structure of adaptive super twisting sliding mode control**. After that, super twisting control is considered

$$\begin{array}{cc} U_{st} & =-{\alpha |\sigma |}^{\frac{1}{2}}\;sign\left(\sigma \right)+v\\ \dot{v} & =-\frac{\beta }{2}sign\left(\sigma \right) \end{array}$$

The adaptive gains are stated as follows:

$$\begin{array}{c} \alpha =\alpha (\sigma ,\dot{\sigma },t) \end{array}$$ (39)

$$\begin{array}{c} \beta =\beta (\sigma ,\dot{\sigma },t) \end{array}$$ (40)

**Lemma 1**. The adaptation law are to be defined as:

$$\begin{array}{cc} \dot{\alpha } & =\{\begin{array}{cc} \omega_{1}\frac{\sqrt{\gamma_{1}}}{\sqrt{2}}\text{sign}\left(|\sigma |-\mu \right), & \text{if}\;\alpha >\alpha_{m}\\ \eta , & \text{if}\;\alpha \leq \alpha_{m} \end{array}\\ \beta & =2\epsilon \alpha \end{array}$$ (41)

The parameters *ϵ*, *γ*_1_, *ω*, *η* are arbitrary positive constants, while *α*_*m*_ is a small positive constant.

Proof. Lyapunov function candidate is introduced as:

$$\begin{array}{c} v\left(\alpha ,\beta \right)=v_{0}+\frac{1}{2\gamma_{1}}{\left(\alpha -\alpha^{*}\right)}^{2}+\frac{1}{2\gamma_{2}}{\left(\beta -\beta^{*}\right)}^{2} \end{array}$$ (42)

where *v*_0_ is Lyapunov function for $\left(\sigma ,\dot{\sigma }\right)$, α*, β* are maximum possible values of *α*, *β* respectively. The derivative of the Lyapunov function is as follows:

$$\begin{array}{c} \begin{array}{cc} \dot{v}\left(\sigma ,\alpha ,\beta \right) & \leq -\eta_{0}\sqrt{v(\sigma ,\alpha ,\beta )}\;-\left|\epsilon_{\alpha }\right|(\frac{1}{\gamma_{1}}\dot{\alpha }-\frac{\omega_{1}}{\sqrt{2\gamma_{1}}})\\ & \;-|\epsilon_{\beta }|(\frac{1}{\gamma_{2}}\dot{\beta }-\frac{\omega_{2}}{\sqrt{2\gamma_{2}}}) \end{array} \end{array}$$ (43)

It gives

$$\begin{array}{c} \dot{v}\left(\sigma ,\alpha ,\beta \right)\leq -\eta_{0}\sqrt{v(\sigma ,\alpha ,\beta )}+\zeta \end{array}$$ (44)

with

$$\begin{array}{c} \zeta =-|\epsilon_{\alpha }|(\frac{1}{\gamma_{1}}\dot{\alpha }-\frac{\omega_{1}}{\sqrt{2\gamma_{1}}})-\left|\epsilon_{\beta }\right|(\frac{1}{\gamma_{2}}\dot{\beta }-\frac{\omega_{2}}{\sqrt{2\gamma_{2}}}) \end{array}$$ (45)

where *ϵ*_*α*_ = *α* − α* and *ϵ*_*β*_ = *β* − β*. It is evident that the system’s finite-time convergence is assured [48].

## 4 Simulation results

The numerical simulation is used to show the efficiency of an improved nonsingular adaptive super twisting sliding mode controller presented in this section. To underscore the importance of this research and the benefits of the proposed controller is compared with those of recently introduced approaches such as normal adaptive SMC in [29], the Super Twisting Sliding Mode Controller (STSMC) in [30], and Adaptive Second Order PID Sliding Mode Controller (ASPIDSMC) [31].

### 4.1 Selection of control parameters

The proposed adaptive controller utilizes the PSO algorithm to fine-tune the gains of the sliding surface. Unlike controllers such as STSMC, ASMC, and ASPIDSMC that rely on fixed gains for the sliding surface, the proposed adaptive controller incorporates adaptive gain tuning and PSO-based optimization to tune all gain values, ensuring that all gains are optimized for the best possible performance. The STSMC utilizes twelve fixed gains for the altitude controller, with gain values determined through trial and error. This approach offers simple implementation due to fixed gains and robustness in certain fixed scenarios but leads to suboptimal performance and limited adaptability to varying conditions. The ASMC utilizes eight gains for the altitude controller, with four of them tuned using an adaptation law. This combination of fixed gains and adaptive tuning improves performance by adjusting some gains and offers better performance compared to controllers with entirely fixed gains. However, the remaining fixed gains can still result in decreased overall performance. The ASPIDSMC utilizes sixteen gains for the altitude controller, with four gains tuned using an adaptation law. This combination enhances the performance and improves disturbance rejection compared to fully fixed gain controllers. In contrast, the proposed adaptive controller continuously adjusts all control parameters using an adaptation law. The PSO algorithm finds the most effective gain values for the sliding surface. This adjustment significantly enhances responsiveness and accuracy, leading to high tracking performance, good disturbance rejection, and smoother control actions.

Table 1, outlines the parameters of quadrotor and Tables 2–4, gives the design parameters of the controller.

**Table 1:** Plant parameter [40].

Plant Parameters	Symbol	Unit	Values
*k*_*f*_	Trust coefficient	*Ns* ^2^	54.2 × 10^−6^
*d*	Drag factor	*Nms* ^2^	1.1 × 10^−6^
*g*	Gravitational Acceleration	*m*/*s*^2^	9.81
*l*	The length of the arm of the quadrotor	*m*	0.24
*m*	Mass of quadrotor	Kg	1
*I*_*xx*_	Inertia around *X*-axis	*Nms* ^2^	8.1 × 10^−3^
*I*_*yy*_	Inertia around *Y*-axis	*Nms* ^2^	8.1 × 10^−3^
*I*_*zz*_	Inertia around *Z*-axis	*Nms* ^2^	14.23 × 10^−3^

**Table 2:** Design parameter for ASTSMC.

Parameter	Value
*α*_*z*_, *α*_*q*1_, *α*_*q*2_, *α*_*q*3_	0.01
*β*_*z*_, *β*_*q*1_, *β*_*q*2_, *β*_*q*3_	0.0002

**Table 3:** Design parameter of adaptation law.

Parameter	Values
*ω*_1_	0.0001
*α*_*m*_	0.01
*η*	0.01
*γ*_1_	2
*μ*	0.7

**Table 4:** Design parameter for PSO SMC.

Parameter	Value
λ_*z*_, λ_*q*1_, λ_*q*2_, λ_*q*3_	80
*c*_*z*_, *c*_*q*1_, *c*_*q*2_, *c*_*q*3_	1

**Remark 1**. The controllers’ design parameters require tuning to attain appropriate performance with regards to quadrotor tracking of trajectory despite disturbances. To determine the optimum values for these parameters, PSO and adaptation laws have been employed.

The simulation results are presented in two distinct phases to effectively illustrate the improved performance of the proposed controller compared to the previously mentioned techniques. During the first phase, spanning from takeoff to fifteen seconds (t = 0 seconds to t<15 seconds,) it is assumed that no external disturbances affect the vehicle. This phase allows for an examination of the tracking performance under ideal conditions. In the second phase, external disturbances are introduced, impacting both the attitude and altitude dynamics of the quadcopter.

### 4.2 Simulation results 1

**Fig 6:**
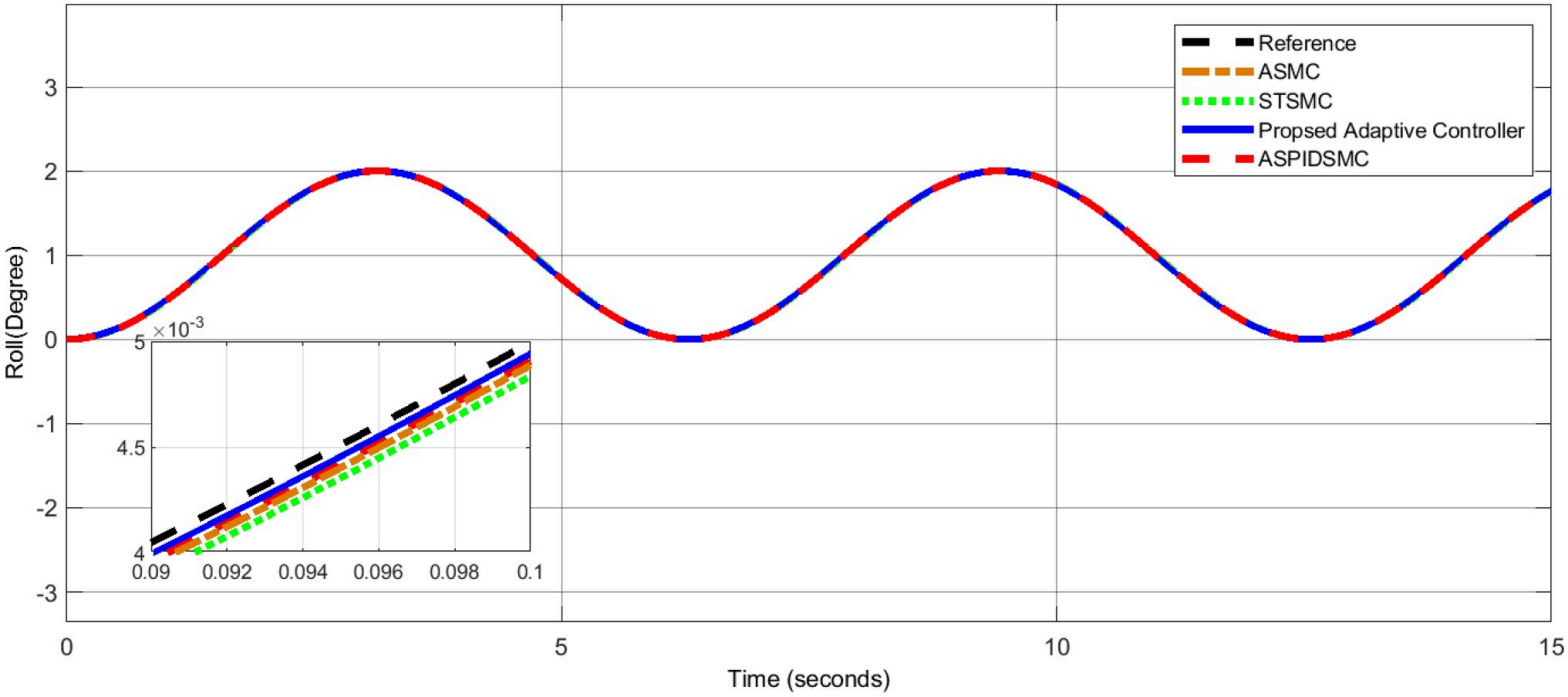
Comparison of roll angle tracking performance between the proposed controller and other controllers.

**Fig 7:**
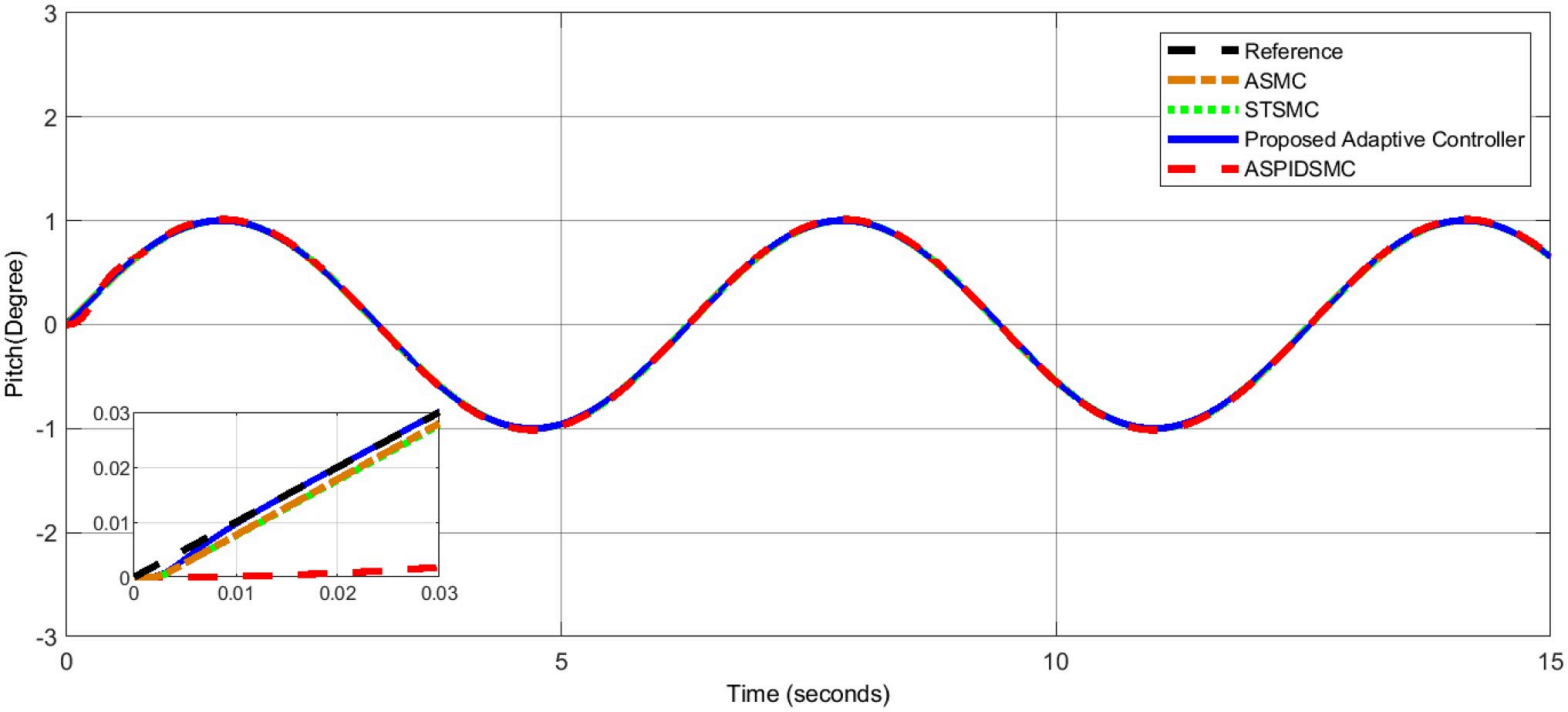
Pitch angle tracking performance, highlighting the tracking response of the proposed controller compared to other controllers.

**Fig 8:**
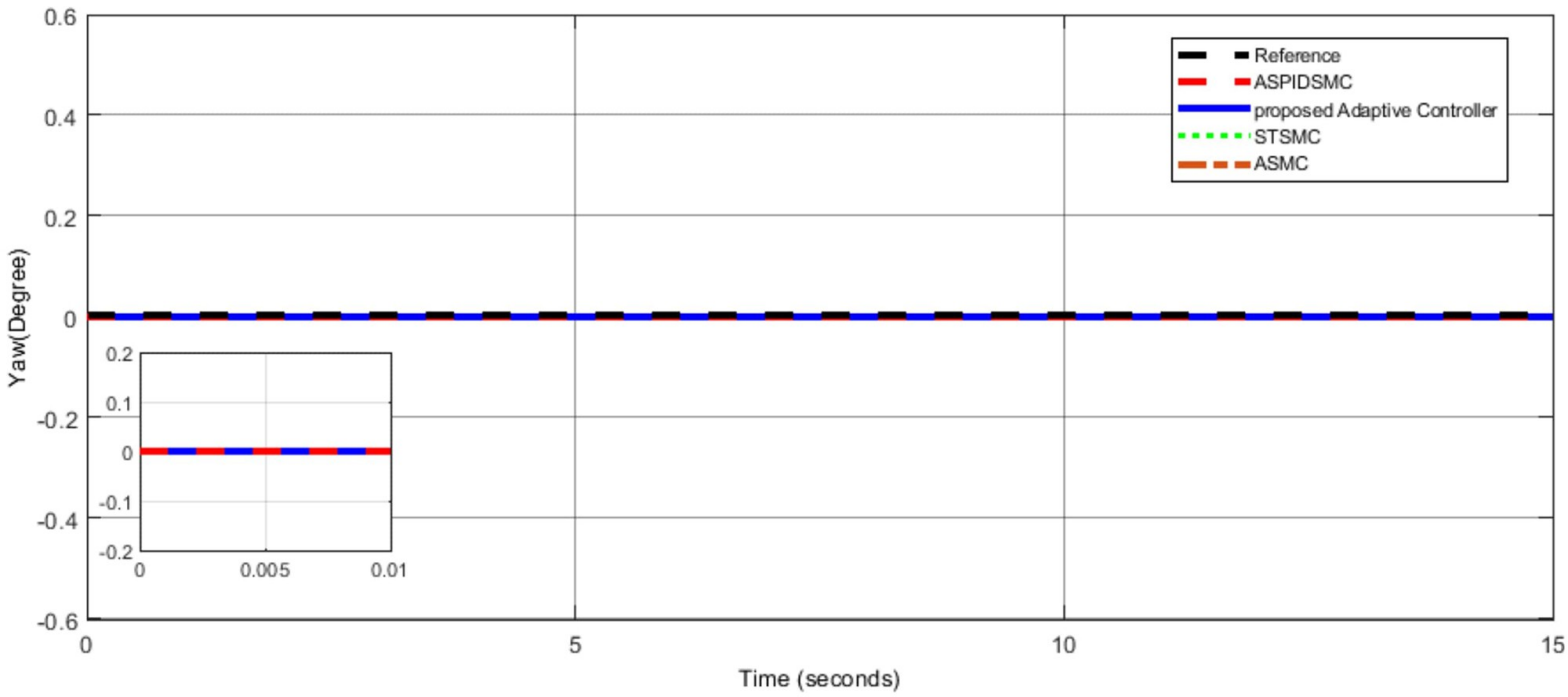
Yaw angle tracking performance comparison, showing similar tracking performance.

**Fig 9:**
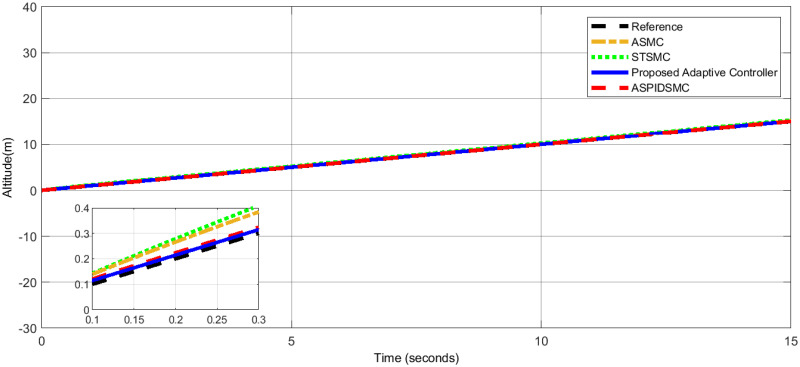
Altitude tracking performance: The proposed adaptive controller shows minimal deviation.

To assess the proposed control scheme’s tracking performance for rotational and translational subsystems the helix input provides the desired path that is employed in this simulation. The performance of three other controller types: ASPIDSMC, STSMC, and conventional adaptive SMC has been simulated and compared with the proposed adaptive controller. It is clear from comparing the simulation results (Figs 6–9), that the proposed controller outperforms the other controllers in terms of tracking performance during the disturbance-free period of time.

The simulation enables testing the controller’s capability to precisely track a dynamic trajectory by using a helical reference input, which shows a circular or helical path for the quadcopter to track. In this paper adaptive technique and PSO is used to obtain the optimal gain for the STSM controller to track the helical trajectory. *ϕ*_*d*_ = 1—cos(t), *θ*_*d*_ = sin(t), and *z*_*d*_ = t.

In Fig 6, the roll angle tracking performance of the quadcopter under different control strategies is presented. The proposed controller demonstrates superior performance in both accuracy and stability. It shows minimal tracking error and quickly tracks the desired roll angle compared to other controllers, such as the STSMC, ASMC, and ASPIDSMC. The roll angle follows the desired trajectory with high precision, indicating the effectiveness of the proposed controller. In Fig 7, depicts the pitch angle tracking performance. Similar to the roll angle results, the proposed controller outperforms the other control methods. It ensures the pitch angle closely follows the desired trajectory with minimal tracking error. The other controllers exhibit larger deviations and slower responses, particularly during the initial transient phase. This highlights the effectiveness of the proposed controller. Fig 8, illustrates the yaw angle tracking performance of the quadcopter under different control strategies.

Fig 9, illustrates the altitude tracking performance. The proposed adaptive controller provides altitude control, maintaining the desired altitude trajectory with minimal deviation. Other controllers, such as the STSMC, ASMC, and ASPIDSMC, exhibit larger initial tracking errors and take longer to stabilize, indicating that the proposed adaptive controller provides more reliable altitude control.

Based on the simulation results, it’s evident that three of the controllers exhibit delays in accurately tracking the trajectory of the quadcopter. To analyze the tracking error of the quadcopter, a numerical method is employed, specifically the ITAE. The utilization of ITAE offers a systematic approach to quantifying the performance of the quadcopter tracking system.

### 4.3 ITAE

ITAE is a performance criterion used in control theory to assess the transient response and tracking performance of control systems. The ITAE is defined as the integral of the absolute error multiplied by time, over a specified time interval. It is given by the formula:

$$ITAE=\int_{0}^{T}t\left|e\left(t\right)\right|dt$$

where:—*e*(*t*) represents the instantaneous error between the desired reference and the actual output at time *t* and *T* is the total duration of the simulation or the time interval of interest. The ITAE metric captures both the magnitude and duration of the error signal, providing a comprehensive measure of tracking performance.

Table 5, presents the ITAE values for tracking performance over 15 seconds. It compares the ITAE metrics for roll, pitch, yaw, and altitude control across different controllers.

**Table 5:** ITAE table for tracking performance for t = 15 seconds.

Parameter	Roll	Pitch	Yaw	Altitude
STSMC	0.4%	1.5%	0	884%
ASMC	0.3%	0.6%	0	175%
ASPIDSMC	0.19%	0.4%	0	7.7%
Proposed adaptive controller	0.1%	0.05%	0	2.2%

### 4.4 Simulation results 2

In subsequent flight phases (t > 5seconds), when disturbances are not zero, the proposed controller outperforms existing techniques. The external disturbances applied at *t* = 5 seconds for both quadrotor position and attitude are specified as follows:

$$\begin{array}{cc} & D_{z}\left(t\right)=0.6\;\text{cos}\left(0.6t\right)+0.7\;\text{sin}\left(0.4\right)N\\ & D_{\text{roll}}=0.1Nm\\ & D_{\text{pitch}}=0.1Nm\\ & D_{\text{yaw}}=0.1Nm \end{array}$$

**Fig 10:**
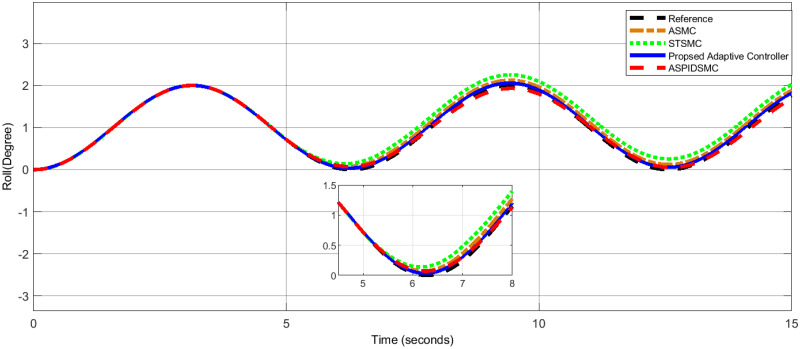
Roll angle performance under disturbances, demonstrating the high disturbance rejection of the proposed controller.

**Fig 11:**
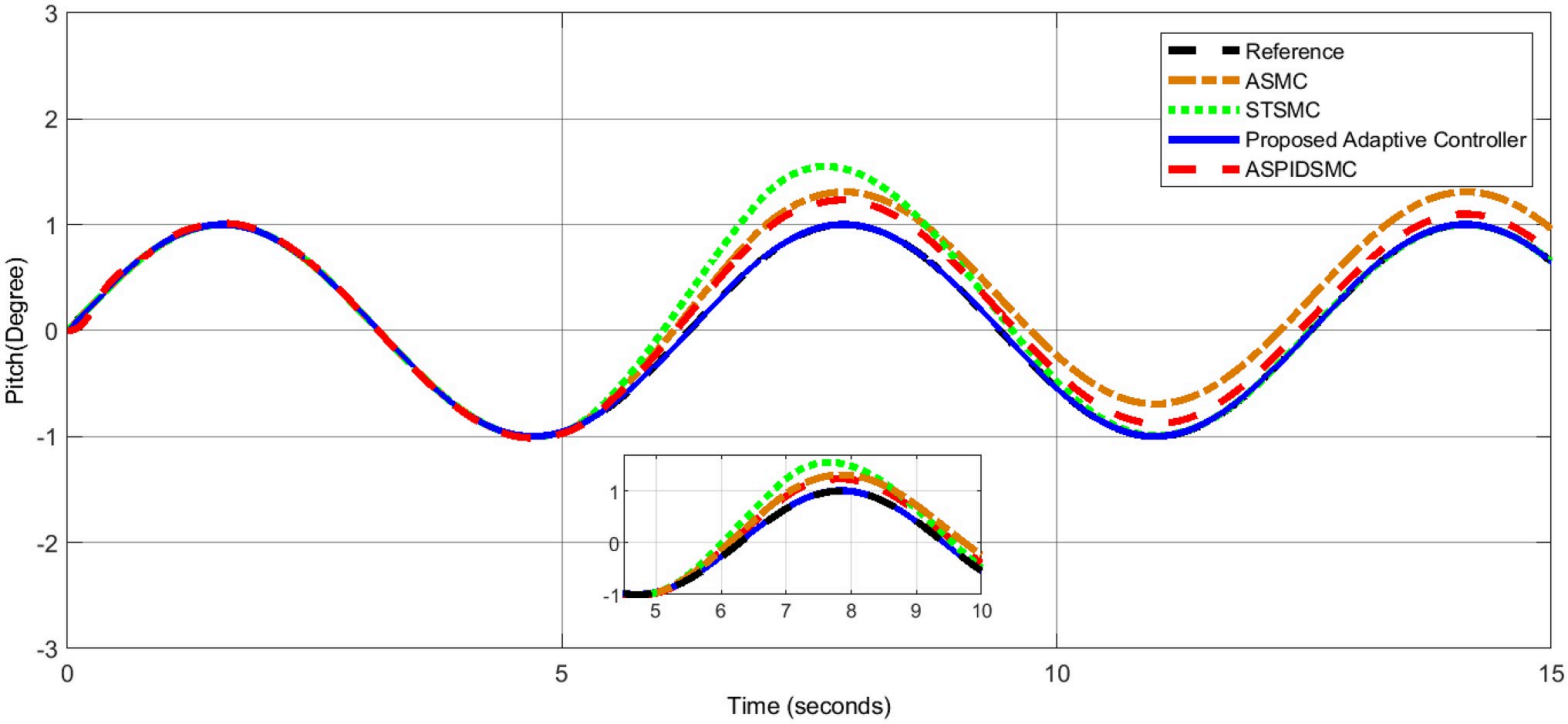
Pitch angle performance, highlighting high disturbance rejection and minimal steady state error of proposed controller.

**Fig 12:**
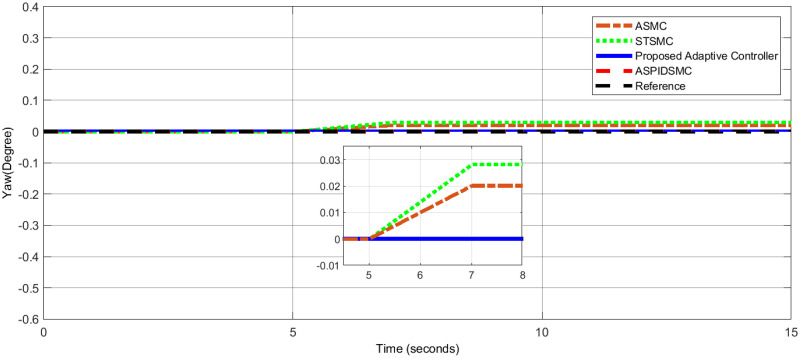
Yaw angle performance under disturbances, highlighting the minimal oscillation of the proposed controller.

**Fig 13:**
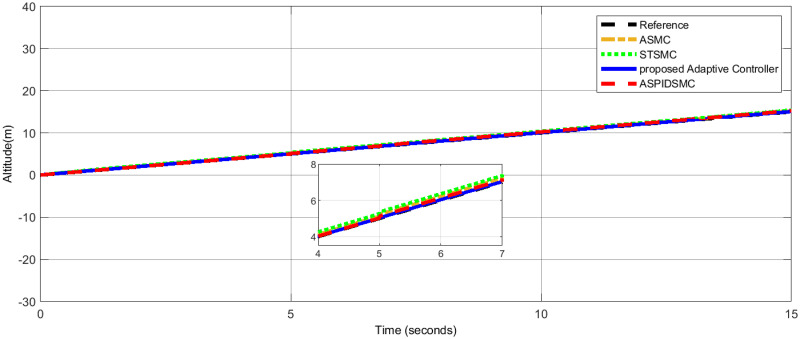
Altitude performance under disturbances, showcasing the high disturbance rejection of the proposed controller.

In Fig 10, the roll angle performance highlights significant differences among the control strategies. The STSMC exhibits substantial oscillations with a steady-state error of approximately 1.2°. The ASMC improves on this with reduced oscillations and a lower error of about 0.5°, due to its adaptive nature. The ASPIDSMC achieves further improvement, reducing the error to around 0.2° due to its enhanced disturbance rejection capabilities. The proposed adaptive controller outperforms all, with a minimal steady-state error of 0.01°, demonstrating superior handling of disturbances and maintaining stability. In Fig 11, the pitch angle performance shows that the STSMC has significant oscillations and an error of about 1.3°, requiring approximately 5.5 seconds to reach a steady state after a disturbance. The ASMC reduces the error to about 0.7°, demonstrating better adaptability and quicker convergence. The ASPIDSMC further refines this to a 0.5° error. The proposed adaptive controller achieves an 0.02° error, quickly restoring stability after a disturbance. Additionally, the STSMC takes around 5.5 seconds to reach the steady state of attitude feedback, while the ASPIDSMC takes about 10.5 seconds for pitch angle. This suggests that the time requires for the vehicle to achieve a steady state after a disturbance occurs is between 5.5 and 10.5 seconds. These results demonstrate how difficult it is for both of these controllers to handle unanticipated disturbances, highlighting the effectiveness of the proposed controller. Fig 12, focuses on yaw angle performance, where STSMC has a minor oscillation with a 0.03° error. The ASMC reduces this to 0.02°, and the ASPIDSMC achieves an impressive 0.002° error. However, the proposed adaptive controller excels with a minimal 0.001° error, highlighting its effectiveness in maintaining yaw stability with minimal oscillations and rapid convergence. Furthermore, in Fig 13, The proposed adaptive controller exhibits better yaw stability, with fewer fluctuations and quicker convergence to the desired yaw angle. The other approaches show more significant oscillations and slower response times, indicating less efficient yaw control. The normal adaptive SMC performs attitude control in the presence of disturbances much better since it switches the SMC on and off using an adaptive law. Although the initial oscillation remains very small, the response of the output never settles to zero over extended period of time, and there is steady state inaccuracy in the roll, pitch, and yaw angles.

**Fig 14:**
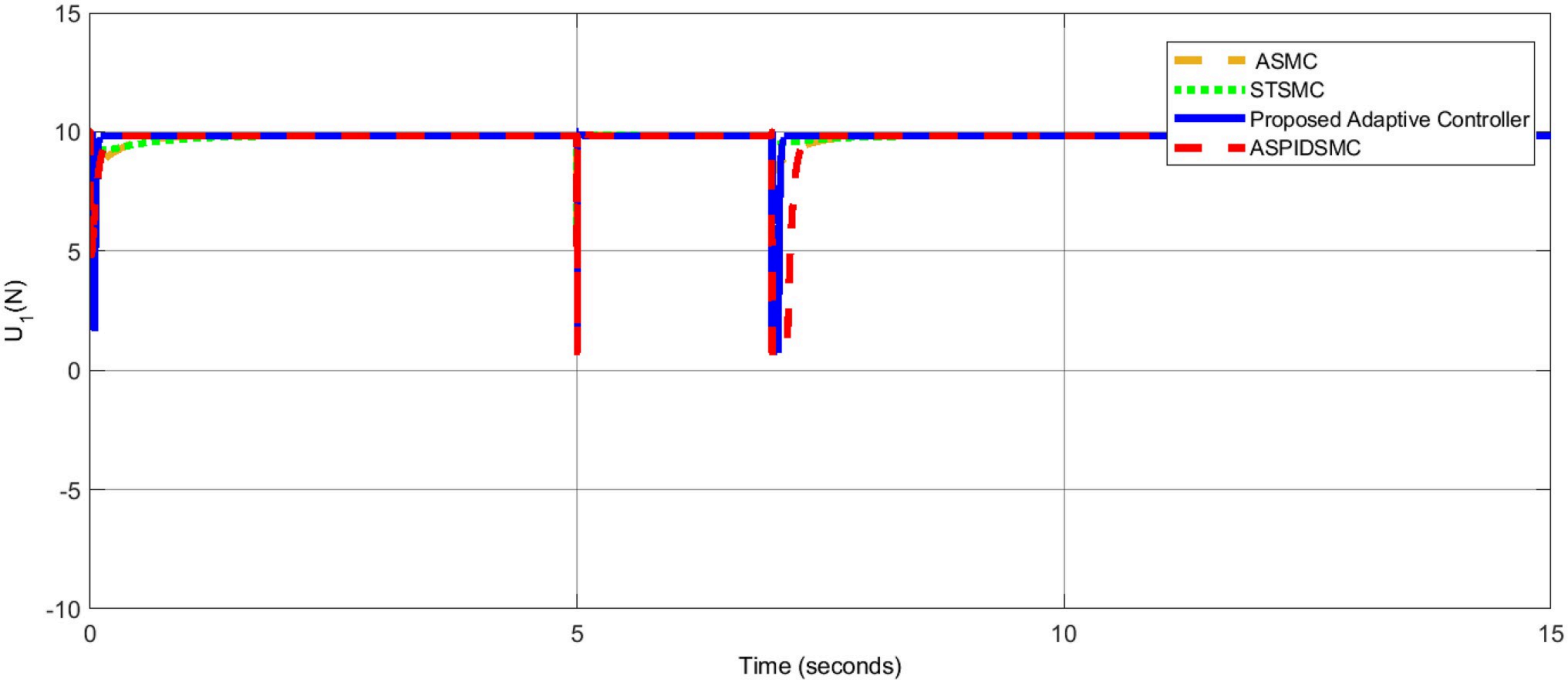
Control effort *U*_1_ for altitude control, demonstrating efficient use of control inputs.

**Fig 15:**
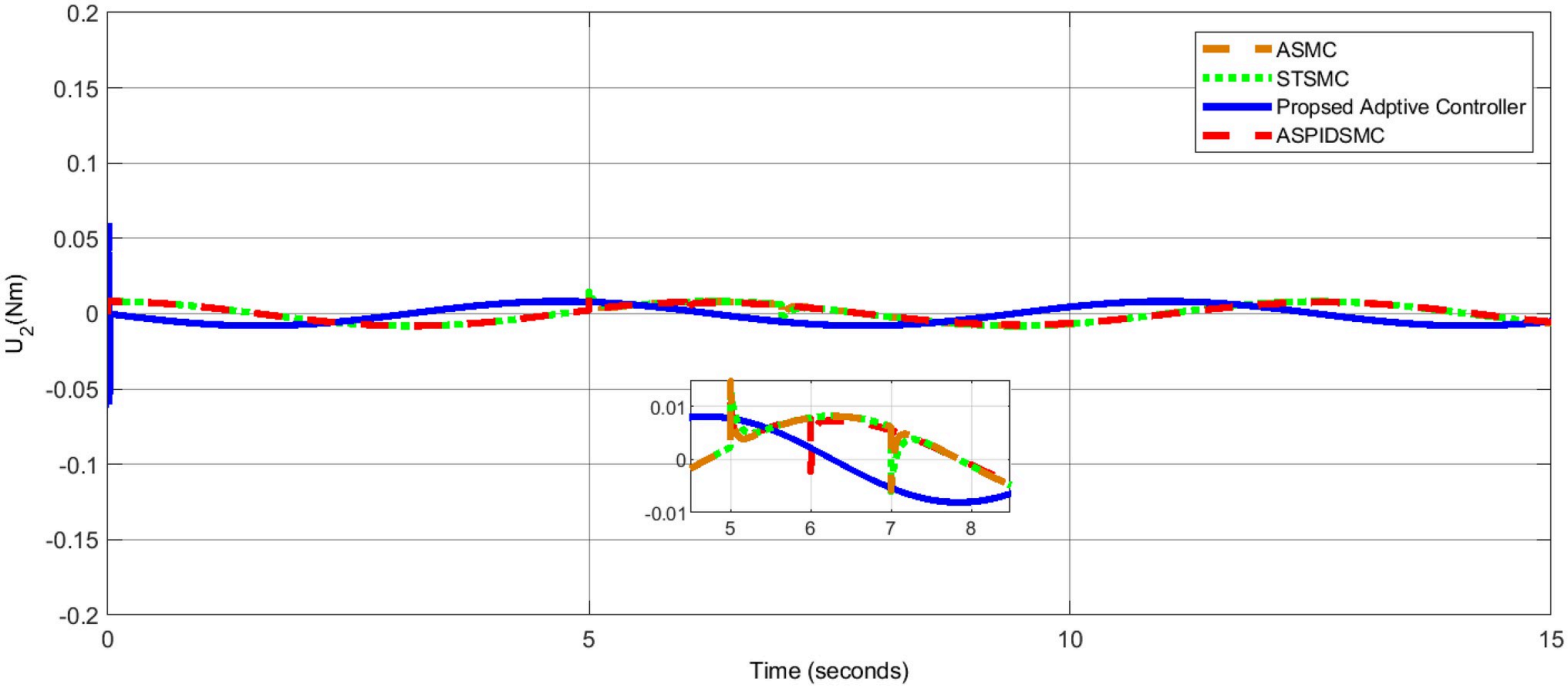
Control effort *U*_2_ for roll motion control, showing minimal and smooth control by the proposed controller.

**Fig 16:**
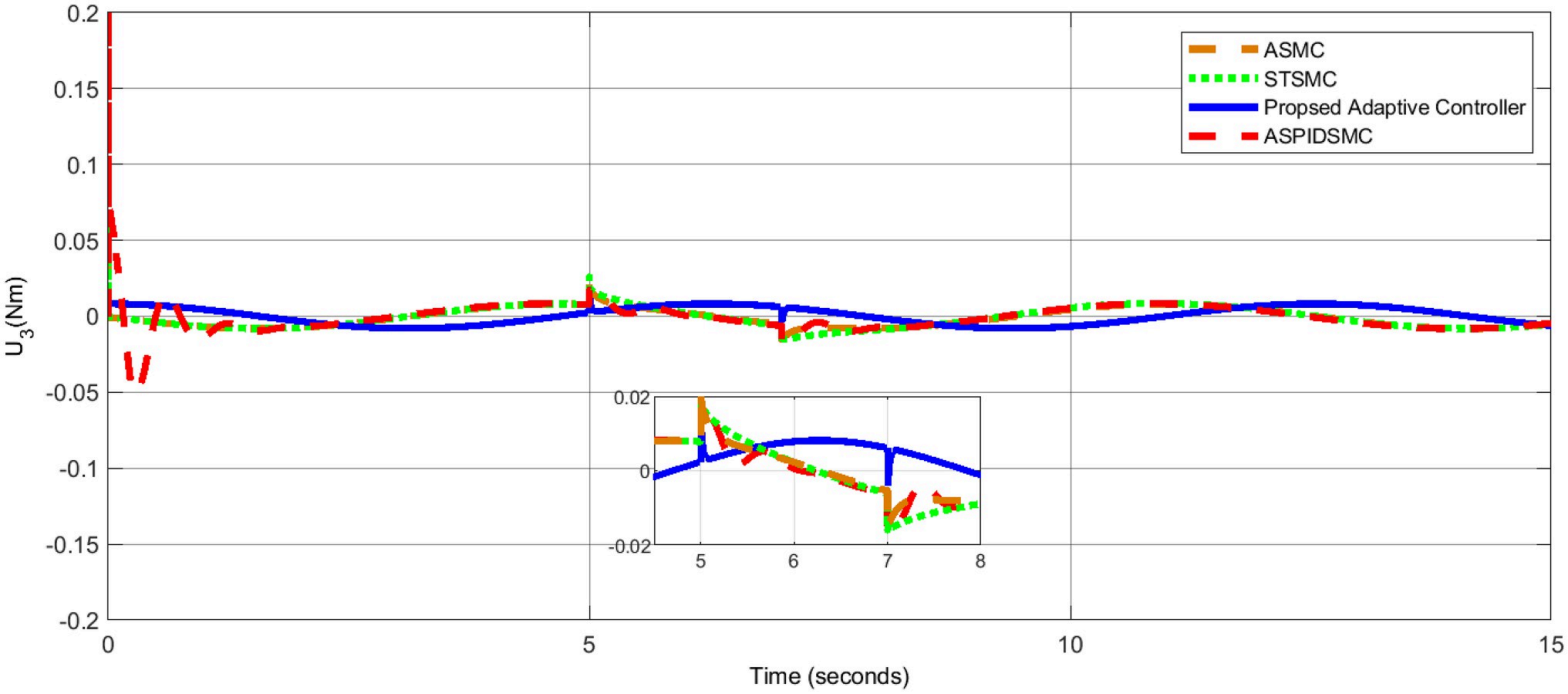
Control effort *U*_3_ for pitch control, showing minimal and smooth effort by the proposed controller.

**Fig 17:**
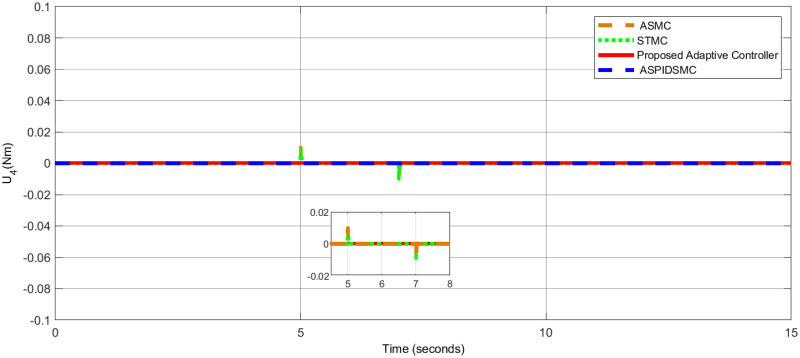
Control effort *U*_4_ for yaw control, highlighting minimal effort by the proposed controller.

Table 6, presents a comparative analysis of the performance of various control methods in terms of roll, pitch, and yaw angles. Each method’s performance is evaluated by the error in degrees for roll, pitch, and yaw angles, with lower values indicating better control performance. The results validate the robustness, precision, and efficiency of the proposed adaptive control strategy for quadrotor UAVs in dynamic and disturbed environments, consistently achieving minimal steady-state errors, rapid convergence, and effective disturbance rejection, outperforming STSMC, ASMC, and ASPIDSMC. Fig 14, shows the control effort *U*_1_ applied to control the quadcopter’s altitude. Fig 15, presents the control effort *U*_2_ required for controlling the roll motion of the quadcopter. Fig 16, depicts the control effort *U*_3_ necessary to control the pitch angle of the quadcopter. Fig 17, illustrates the control effort *U*_4_ necessary to control the yaw motion of the quadcopter.

**Table 6:** Results of control performance.

Parameter	Roll	Pitch	Yaw
STSMC	1.2°	1.3°	0.03°
ASMC	0.5°	0.7°	0.02°
ASPIDSMC	0.2°	0.5°	0.002°
Proposed adaptive controller	0.01°	0.02°	0.001°

On the other hand, the proposed adaptive controller in this paper demonstrates a short oscillation that ends rapidly as the steady state is restored. The controller appears to be able to instantly reduce the effect of the external disturbance once it is applied. As a result, there is very little oscillation in attitude (0.01° for pitch and roll and 0.001° for yaw) is formed. The performance of the other tested controllers at altitude when disturbances occur (t > 5seconds), the quadcopter altitude performance is significantly impacted, exhibiting relatively large oscillations in comparison to the proposed controller. Moreover, once the disturbance has been applied to the vehicle, STSMC and ASPIDSMC similarly show a longer delay before convergent to the steady state. It appears that the suggested adaptive controller performs excellent performance, with good tracking and the ability to reject disturbances, demonstrating the effectiveness of our suggested algorithm for altitude control. In summary, the results of the simulation demonstrated that all four of the tested systems demonstrated there were noticeable differences in performance as soon as the system was subjected to outside disturbances. Due to their slow adaptation to these disturbances, the roll, pitch, yaw, and altitude oscillations were comparatively large for STSMC, ASMC, and ASPIDSMC.

For *U*_1_, *U*_2_, *U*_3_, and *U*_4_, the control efforts are 9.81*N*, 0.03*Nm*, 0.02*Nm* and 0.01*Nm*, respectively. In contrast to the other controller, the control actions that are taken with low effort are executed smoothly. These show that the control algorithm developed is effective. When comparing the three controller control efforts that are feasible in presence of external disturbance minimum control effort is expressed clearly. As a result, the proposed control strategy stabilizes the quadrotor despite the loss of optimal control efforts to guarantee accurate tracking of the required reference model trajectory.

## 5 Conclusions

A non-singular adaptive super twisting sliding mode controller that controls the quadrotor position and attitude is developed and tested. In order to develop the proposed controller, first the flight dynamics of quadrotor is modeled taking into account all the phenomena that are relevant for the maneuvering of the quadrotor UAV. Subsequently, the proposed controller is designed for performance comparison, with ST SMC, ASMC, and ASPIDSMC. Finally, the efficiency of the proposed control method has been confirmed using numerical simulations in both normal and disturbed environments. The results of the simulation indicate the effectiveness of the proposed control method attaining excellent accuracy in tracking and a high capacity to reject disturbances. Moreover, the desired results can be achieved with minimal and smooth control actions. These show the cost-effectiveness and functional safety of the complete control architecture, suggesting that the proposed controller may be deployed in real time for the quadrotor platform.
